# Effect of a lifestyle intervention program's on breast cancer survivors' cardiometabolic health: Two-year follow-up

**DOI:** 10.1016/j.heliyon.2023.e21761

**Published:** 2023-10-29

**Authors:** Valentina Natalucci, Carlo Ferri Marini, Francesco Lucertini, Giosuè Annibalini, Davide Sisti, Luciana Vallorani, Roberta Saltarelli, Andrea Rocco Panico, Marta Imperio, Marco Flori, Paolo Busacca, Anna Villarini, Sabrina Donati Zeppa, Deborah Agostini, Silvia Monaldi, Simone Barocci, Vincenzo Catalano, Marco Bruno Luigi Rocchi, Piero Benelli, Vilberto Stocchi, Elena Barbieri, Rita Emili

**Affiliations:** a Department of Biomolecular Sciences, University of Urbino Carlo Bo, 61029 Urbino, Italy; b U.O.C. Cardiologia/UTIC, AST, Ospedale Santa Maria della Misericordia, 61029, Urbino, Italy; c Department of Medicine and Surgery, University of Perugia, Piazzale Settimio Gambuli, 06132, Perugia, Italy; d U.O.C. Oncologia Medica, AST, Ospedale Santa Maria della Misericordia, 61029, Urbino, Italy; e U.O.C. Patologia Clinica, AST, Ospedale Santa Maria della Misericordia, 61029, Urbino, Italy; f Department of Human Sciences for the Promotion of Quality of Life, University San Raffaele, 20132, Roma, Italy

**Keywords:** Breast cancer survivors, Home-based lifestyle intervention, Aerobic exercise, Mediterranean diet, COVID-19

## Abstract

The purpose of this study is to assess the cardiometabolic responses of a lifestyle intervention (LI) conducted at home among breast cancer (BC) survivors during the two years of COVID-19 pandemic. A 3-month LI focused on diet and exercise was performed on thirty BC survivors (women; stages 0-II; non-metastatic; aged 53.6 ± 7.6 years; non-physically active) with a risk factor related to metabolic/endocrine diseases. Anthropometrics, cardiorespiratory fitness ($\dot{V}$*O*_*2max*_), physical activity level (PAL), adherence to the Mediterranean diet (MeDiet modified questionnaire), and several biomarkers (i.e., glycemia, insulin, insulin resistance [HOMA-IR] index, triglycerides, high- [HDL] and low- [LDL] density lipoproteins, total cholesterol, progesterone, testosterone, and hs-troponin) were evaluated before and 3-, 6-, 12-, and 24-month after the LI. Beneficial effects of the LI were observed on several variables (i.e., body mass index, waist circumference, MeDiet, PAL, $\dot{V}$ O_2max_, glycemia, insulin, HOMA-IR index, LDL, total cholesterol, triglycerides, testosterone) after 3-month. The significant effect on Mediterranean diet adherence and $\dot{V}$ O_2max_ persisted up to the 24-month follow-up. Decreases in HOMA-IR index and triglycerides were observed up to 12-month, however did not persist afterward. This study provides evidence on the positive association between LI and cardiometabolic health in BC survivors.

## 1 Introduction

Breast cancer (BC) is the most common global malignancy and the leading cause of cancer deaths among women worldwide [1]. However, thanks to breast screening and more effective BC treatments, the 5-year relative survival rate improved (88 %) [2,3] with an estimated BC survivors in Europe at 2 millions it is expected to increase in the coming decades [4]. Particularly, in the window of survival the focus is twofold: on the one hand, it is aimed at improving the quality of life; on the other hand, at reducing potential risk factors (e.g., increased waist circumference, elevated triglycerides, hypertension, high fasting glucose levels, and low high-density lipoprotein (HDL), cholesterol levels), which are considered risk factors of mortality and recurrence. In this regard, an increasing number of survivors experience strong motivation to improve their general health after diagnosis [5]. As some studies emphasize, paying constant attention to patients with cancer after diagnosis, during and after treatments is useful to help maintain proper nutrition and adequate physical activity levels (an healthy lifestyle) over time [6,7]. Considering the influence of lifestyle factors on the risk of recurrence and/or death after BC diagnosis [8], more attention should be paid to patients at higher risk of BC recurrence, such as sedentary patients and those affected by metabolic disorders. In agreement with the National Comprehensive Cancer Network (NCCN) and the World Health Organization (WHO), identifying the lifestyle factors related to development of BC recurrence and their influence on health-related quality of life in this population represents an emerging medicine approach [9,10]. Indeed, new evidence suggests that lifestyle interventions, which include exercise and proper nutrition, are critical in the process of overcoming the BC disease. The beneficial effects induced by exercise are commonly attributed to physiological adaptations over time (i.e., months/years) and are related to improvements in both cardiometabolic outcomes (e.g., increased cardiorespiratory fitness, and reduced inflammation and overall mortality) and health-related outcomes (e.g., depressive symptoms, fatigue, and quality of life) [[11], [12], [13]]. These beneficial effects result from the implementation of the exercise guidelines for cancer survivors at the end of primary treatments (i.e., chemotherapy and/or radiotherapy). As recommended by the American College of Sports Medicine (ACSM), cancer survivors should engage in at least 150–300 min/week of moderate intensity aerobic training and include resistance training exercise at least 2 days per week. Particularly, exercise prescription as an adjuvant therapy of cancer patients includes moderate-intensity aerobic exercise at least three times per week, for at least 30 min, for at least 8–12 weeks [10,14]. In addition to exercise, diet is also an essential pillar of a healthy lifestyle. An overall healthy dietary pattern has the potential to lower cancer risk and progression and, these benefits have also been outlined in the WCRF/AIRC 2018 recommendation [[15], [16], [17], [18], [19]]. These recommendations focus on a diet rich in whole grains, vegetables, fruits and beans, limited consumption of processed foods high in fats, starches or sugars, red and processed meat, beverage sugary foods, alcohol and the lack of use of supplements [19]. Currently, the Mediterranean diet rich in whole grains, vegetable, and legumes seems to be a dietary pattern able to reflect many characteristics of an ideal healthy diet [[15], [16], [17], [18]]. The properties of these foods reduce the risk of metabolic syndrome, which is one of the risk factors for BC. In addition, preferring unrefined foods and limiting animal fats is another healthy advice because these tend to slow down the action of insulin and decrease glycemia, both factors associated with greater risk of developing cancer or incurring relapses [19,20]. Despite the importance of maintaining a healthy lifestyle after BC treatments, few studies have reported longer-term follow-up data on cardiometabolic risk factors after lifestyle intervention based on exercise and/or nutrition. Although this study was conducted in an emergency period (i.e., along the 2-Year COVID-19 pandemic), it is possible to consider that the adapted lifestyle intervention could provide interesting strategies to help BC survivors. During the past 3 years, the impact of the COVID-19 pandemic has resulted in the application of alternative strategies for prescribing and monitoring healthy lifestyle (i.e., exercise and proper nutrition) by health professionals [21,22] which allowed us to reflect on other strategies implemented over the years with medium-long term effects [7,23]. In this regard, it is interesting to note that technology has the promise of being an effective method of monitoring physical activity for prevention, treatment and improvement of health and quality of life in people with non-communicable diseases such as a patient with cancer [24,25]. However, considering current European and Global health and fitness trends, further efforts are needed to understand their applicability in terms of prescribing a lifestyle program [26,27]. In the light of the preliminary short-term (3-month) results reported by Natalucci et al. [28], the aim of this study is to describe the short- (3- and 6-month) and long-term (12- and 24-month) impact of a lifestyle intervention based on exercise and nutrition for BC women at high risk of recurrence due to metabolic/endocrine diseases during two years since the beginning of the COVID-19 pandemic.

## 2 Materials and methods

### 2.1 Setting and participants

The methods of the MoviS Trial have been reported elsewhere [29]. Briefly, the MoviS Trial was a monocentric trial conducted at the Santa Maria della Misericordia Hospital in Urbino and the Department of Biomolecular Sciences of University of Urbino Carlo Bo in the Marche region (central Italy). Ethical approval was granted from the Human Research Ethics Committee of the University of Urbino Carlo Bo (Protocol N 21 of July 10, 2019) and the MoviS trial was registered (protocol: NCT 04818359) [29]. Written informed consent was obtained from all participants. Participants were eligible for the study if they were women with histologically confirmed BC (stage 0-III) with no evidence of recurrent or progressive disease at recruitment; were within 12-month after surgery, and minimum 6-month after radiotherapy, and/or chemotherapy with or without current hormone therapy use; were aged between 30 and 70 years; were at risk of recurrence for at least 1 of the following conditions: body mass index (BMI) ≥ 25 kg/m^2^; testosterone ≥0.4 ng/mL; serum insulin ≥25 μU/mL (170 pmol/L); and metabolic syndrome. Women were excluded in case of recurrent disease, physically active in the previous 6 months, or if they had pneumological, cardiological, neurological, orthopaedic comorbidities, or mental illnesses that prevent the exercise performance [29].

### 2.2 Study design and amended protocol

**Fig. 1:**
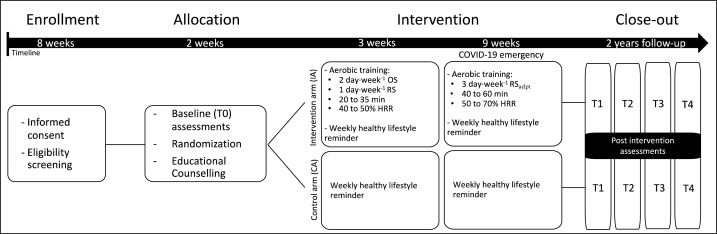
Study design flowchart. HRR, heart rate reserve; on-site (OS) and remotely (RS) supervised; RS_adpt_, adapted remote supervision due to COVID-19 pandemic; follow-ups after 3 (T1), 6 (T2),12 (T3), and 24 (T4) months.

The study was an open randomized controlled trial with two parallel groups (1:1 randomization ratio with the control arm). The randomization was achieved using the randomized block permutation method (n = 4) to ensure balance between the two groups. Randomization lists were generated using an Excel spreadsheet. Briefly, patients were randomly assigned to the intervention arm (IA) (lifestyle recommendations and MoviS training) or the control arm (CA) (lifestyle recommendations). As reported by Natalucci et al. [28] due to the Italian restriction from COVID-19 resulted in a modification of the original protocol [29]. The amendment was approved by the Human Research Ethics Committee of the University of Urbino Carlo Bo (Protocol N 29 of 22 April 2020) (Fig. 1). Forced changes in study protocol made the difference on cardiometabolic parameters negligible between the IA and the CA, providing similar adjustments between groups. Indeed, previous multivariate and univariate effects of the interaction between time (baseline and post-intervention) and study arm (IA and CA) showed that the differences on several representative parameters' interaction were not significative (please, see Supplementary Table S1 by Natalucci et al. [28]). Additionally, as reported in the Supplementary Table S1, the two groups show no significant differences at T1 in any of the analyzed parameters. For these reasons, the two arms (IA and CA) were combined. More details on the modification of the study design are presented in the ‘Intervention description’ paragraph.

### 2.3 Intervention description

Lifestyle intervention included exercise and nutritional recommendations for both arms (IA and CA) while, a targeted exercise intervention, the MoviS Training, was carried out only in the participants of the IA. Specifically, during a meeting lasting about an hour (45 min group and 15 min personalized), an oncology nutritionist and an exercise oncology specialist, presented and discussed with both arms participants the current guidelines for exercise and Mediterranean diet. They received recommendations suggested by the WCRF 2018 and the recent nutritional and exercise guidelines for patients with BC, approved by the Ministry of Health 2017 and 2019 [19,30,31]. In addition, both arms were given the opportunity to register on the DianaWeb platform [32,33] through which the participants could find daily nutritional advice based on Mediterranean diet, while only the IA received the MoviS training. The MoviS Training included three months of aerobic exercise training with a gradual increase in exercise intensity (40–70 % of heart rate reserve [HRR]) and duration (2 days/week of directly supervised exercise and 1 day/week of remotely monitored exercise) (20–60 min). Heart rate responses were tracked during the training sessions, and external exercise intensities (such as bike power or treadmill speed and grade) were changed to keep the target HRR constant despite the numerous physiological changes that occur during protracted aerobic activities (e.g., cardiovascular drift) [34]. Restrictions related to the COVID-19 epidemic forced the exercise supervision in presence to change to only remotely supervised exercise starting in the fourth week (3 sessions per week). Participants in the IA received weekly phone calls from the exercise specialist, who provided the prescription and personalized feedback according to the training logs. For each exercise session, each participant could choose the exercise modality based upon the restriction and the availability of training devices such as treadmills or bikes in the home, while exercise intensity was always prescribed and monitored by exercise specialist using heart rate. Indeed, each participant was provided with a heart rate monitor (ONRHYTHM 110, Kalenji) and instructed on how to use it to reach the target exercise intensity and monitor the exercise sessions independently. Additional information is reported by Natalucci et al. [28].

The usual care received by participants in the CA during the intervention phase consisted of lifestyle recommendations (dietary recipes, exercises, and suggestions) to manage improvements in their daily routine based on nutrition and physical activity guidelines [10,14,35] and were remotely supported using group chat messages in a random weekday and time. The study design is represented in Fig. 1.

### 2.4 Assessments

#### 2.4.1 Anthropometrics and body composition

A fixed stadiometer was used to record height to the nearest 0.5 cm, and an electronic scale was used to measure body weight to the nearest 0.1 kg while the patients wear a hospital gown and without shoes. A fixed stadiometer was used to record height to the nearest 0.5 cm, and an electronic scale was used to measure body weight to the nearest 0.1 kg while the patients wear a hospital gown and without shoes. The midpoint between the iliac crest and the lower edge of the last palpable rib was used to estimate waist circumference. Measurements of bioimpedance were made using the DC430MA DC430 (Tanita Europe).

#### 2.4.2 Dietary habits and physical activity level

The Mediterranean Diet was assessed by using the MeDiet modified questionnaire, which participants filled out through the DianaWeb Platform (Supplementary Table S2) [36]. The MedDiet score was used to measure adherence to the Mediterranean diet. Physical activity level was assessed by using the SenseWear armband (BodyMedia Inc., Pittsburgh, PA), a multisensory monitor worn on the back of the upper arm (over the triceps muscle) as per manufacturer orientation. Participants wore the sensor for at least 5 consecutive days (24h a day).

#### 2.4.3 Cardiorespiratory fitness

Submaximal cardiorespiratory fitness tests were performed at every assessment time. The submaximal incremental exercise testing procedures have been described in detail by Natalucci et al. [28]. Briefly, personalized tests were created according to the predicted $\dot{V}$ O_2max_ of the participants. The tests started at an exercise intensity (estimated from treadmill speed and grade using the ACSM's metabolic equation for walking equation [35]) corresponding to approximately 30 % of the estimated oxygen uptake ($\dot{V}$ O_2_) reserve ($\dot{V}$ O_2_R). Then the exercise intensity was increased of approximately 10 % of $\dot{V}$ O_2_R every 3 min until 70 % of HRR was reached [37]. The HR and $\dot{V}$ O_2_ corresponding to the desired percentages of the reserve values (i.e., R and HRR) were calculated using the following formula: (maximal value – resting value) x desired percentage + resting value. The resting $\dot{V}$ O_2_ was estimated to be 3.5 mL min^−1^ kg^−1^ [35], whereas $\dot{V}$ O_2max_ was predicted by means of a non-exercise model using the Excel file provided by Ferri Marini et al. [38]. The resting HR was measured after the participants sat quietly for 10 min, whereas the maximal HR (HR_max_) was estimated according to participants' age [39]. The $\dot{V}$ O_2max_ of each participant was then estimated based upon her HR responses at each stage of the test by extrapolating the individual submaximal HR-$\dot{V}$ O_2_ relationship to the predicted HR_max_ [36,37]. The individualized submaximal incremental testing protocols created at baseline were repeated at each follow-up.

#### 2.4.4 Biomarkers

Blood samples baseline and after 3-, 6-, 12- and 24-month were obtained following a 12-h fast from the antecubital vein by trained phlebotomists no-exercise. All blood samples were collected into 10 ml EDTA, sodium heparin, or serum separator vacutainer tubes. An enzyme-linked colorimetric test was used to measure blood glucose on a clinical chemistry analyzer (Beckman Coulter AU Analysers); an automatic analyzer used a competitive binding test was used to determine the levels of serum insulin; whereas plasma triglycerides and cholesterol were assessed using a Cobas Integra 400 and a Beckman Coulter DXC800. With intraday and interday variability of 2 %, all assays met manufacturers’ guidelines [40,41]. HOMA-IR index was calculated as reported in Ref. [42]: fasting insulin × fasting glucose (mmol/L)/22.5). The other hormonal markers such as progesterone, estradiol and testosterone were measured using the chemiluminescence Beckman Coulter DXi analyzer as reported in Refs. [43,44].

### 2.5 Statistical analyses

Statistical analyses were performed to assess if the BC survivors showed differences on the variables considered in this study, over two years. For each variable, the mean, the standard deviation (SD), and the percentage of change (% of change) during the follow-up (T1, T2, T3, and T4) relative to the baseline values were calculated. In this study, a per-protocol analysis was used, and the missing data were not imputed. The clinical and functional parameters analyzed were the following: body weight, BMI, waist circumference, fat mass, cardiorespiratory fitness, physical activity level, adherence to Mediterranean diet, and several biomarkers (i.e., glycemia, insulin, HOMA-IR index, triglycerides, HDL, LDL, total cholesterol, progesterone, testosterone, and hs-troponin). The outcomes were analyzed using repeated measures MANOVA (multiple analysis of variance) models, with differences-based contrasts for each dependent variable (reference was measured at T0). Analysis was performed using IBM SPSS Statistical Software version 27.0.

## 3 Results

### 3.1 Subject characteristics

Thirty BC survivor women were enrolled (baseline clinical characteristics are shown in Table 1). At baseline no differences were found in demographic and basal clinical data between the two arms (Supplementary Table S1). All participants met the inclusion criteria and, in average, were overweight (Table 2). At 24-month post-intervention the following adverse events were observed: one relapse between 12- and 24-month, and one dropout between the third and sixth month due to knee cartilage damage. No cardiovascular incidents were observed over time. The adherence to training in % session (computed as the percentage of the sessions performed at the prescribed intensity and duration) [29] ranged from 63.9 % to 100 % among the IA participants, with an average of 88.9 % for the total sample. All participants had an average adherence rate >50 %, and ten achieved average adherence rates >80 %.

**Table 1:** Baseline characteristics of the study sample (n = 30).

	Overall (n = 30)
Age (y)^a^	53.6 ± 7.6
Stage, n (%)	
0	6 (20)
I	15 (50)
II	9 (30)
III	–
Menopausal status, n (%)	
Postmenopausal	18 (64.3)
Surgery Type, n (%)	
Mastectomy	3 (10.0)
Quadrantectomy	26 (87.7)
Lumpectomy	1 (3.3)
Treatment in addition to surgery, n (%)	
Only radiation	2 (6.7)
Only chemotherapy	13 (43.3)
Radiation and chemotherapy	4 (13.3)
None	11 (36.7)
Current endocrine therapy, n (%)	
None	6 (20.0)
Tamoxifen	8 (26.7)
Aromatase Inhibitor	16 (53.3)

Abbreviations: n, number of participants; %, percentage of total number of participants.

a Data are presented as mean ± SD.

**Table 2:** Comparison of physical activity level, dietary habits, and anthropometric and, cardiorespiratory fitness parameters at T0 vs T1, T2, T3, and T4, along with their percent changes at T1, T2, T3, and T4 vs T0.

	Mean ± SD (T0)	Mean ± SD (T1)	Mean ± SD (T2)	Mean ± SD (T3)	Mean ± SD (T4)	% of change T1 vs T0	% of change T2 vs T0	% of change T3 vs T0	% of change T4 vs T0
**Body weight (kg)**	67.58 ± 11.51	66.82 ± 10.73	67.10 ± 11.43	67.44 ± 11.85	68.16 ± 10.90	−1.13 %	−0.70 %	−0.21 %	0.85 %
**BMI (kg/m**^**2**^**)**	26.17 ± 4.97	25.69 ± 4.62*	26.07 ± 4.77	26.00 ± 5.05	26.24 ± 4.73	**−1.83 %**	−0.37 %	−0.65 %	0.28 %
**Waist Circumference (cm)**	85.36 ± 11.37	85.00 ± 10.69	82.40 ± 10.99*	86.00 ± 12.36	85.64 ± 11.29	0.30 %	**−3.46 %**	0.75 %	0.33 %
**Fat mass (%)**	21.73 ± 8.08	21.12 ± 7.50	21.77 ± 8.40	21.97 ± 8.86	23.05 ± 8.01*	−2.81 %	0.17 %	1.13 %	**6.10 %**
$\dot{V}$ O_***2max***_**(mL·min**^**−1**^**kg**^**−1**^**)**	30.50 ± 5.65	33.92 ± 6.44***	33.88 ± 6.38***	32.70 ± 5.89**	32.65 ± 5.46**	**11.21 %**	**11.08 %**	**7.19 %**	**7.04 %**
**PAL (MET-min/week)**	1.39 ± 0.24	1.42 ± 0.24	1.34 ± 0.27	1.41 ± 0.27	1.33 ± 0.21*	1.93 %	−3.72 %	1.22 %	**−4.51 %**
**Adherence to Mediterranean diet (MeDiet Score DianaWeB)**	6.72 ± 1.85	7.93 ± 2.01***	8.24 ± 1.93***	8.31 ± 1.87***	7.97 ± 1.36**	**17.95 %**	**22.62 %**	**23.69 %**	**18.62 %**

Abbreviations: BMI, body mass index; $\dot{V}$ O_2max_, maximal oxygen uptake; PAL, physical activity level; T0, baseline; after 3 (T1); 6 (T2); 12 (T3), and 24 (T4) months after the lifestyle intervention; bold values denote statistical significance with a p < 0.05 (*); p < 0.01 (**); or p < 0.001 (***).

### 3.2 Changes in anthropometric, body composition, cardiorespiratory fitness parameters, physical activity level, dietary habits

Anthropometric measurements of all the participants are listed in Table 2. BMI was significantly reduced by −1.83 % immediately after 3-month of lifestyle intervention (T1 vs. T0, p = 0.032), however, it was not maintained at follow-up points T2, T3 and T4. Waist circumference was maintained similar between T0 and T1 and was significantly reduced by −3.46 % in BC survivors (T2 vs. T0, p = 0.002), but did not persist at the other follow-up points, and body weight slightly decreased by −1.13 % after 3-month of lifestyle intervention (T1 vs. T0 without significance). Fat mass remains fairly stable over the follow-up times, with a slight decrease after 3-month of lifestyle intervention and an increase at 24-month of follow-up (−2.81 % T1 vs. T0, no differences between T2 and T0; +1.13 % T3 vs. T0, +6.10 % T4 vs. T0, p = 0.036). Based on submaximal incremental walking test, the cardiorespiratory fitness improved significantly at T1 and values were maintained over the different time points. The $\dot{V}$ O_2max_ values from the baseline increased in BC survivors by +11.21 % (T1 vs. T0, p < 0.001), +11.08 % (T2 vs. T0, p < 0.001), +7.19 % (T3 vs. T0, p = 0.002), +7.04 % (T4 vs. T0, p = 0.019). Physical activity level did not change significantly over time except at T4 (−4.51 % T4 vs. T0, p = 0.036). The increase of adherence to the Mediterranean diet was significant and, as shown in Table 2, persisted through follow-up points (+17.95 % T1 vs. T0, p < 0.001; + 22.62 % T2 vs. T0, p < 0.001; + 23.69 % T3 vs. T0 p < 0.001 and + 18.62 % T4 vs. T0 p = 0.002, respectively). The study found that improvements in $\dot{V}$ O_2max_ and adherence to the Mediterranean diet were sustained up to two years of follow-up, whereas the short-term improvements in BMI and waist circumference were not maintained two years after the intervention. The fat mass and physical activity level remained stable in the short term, but they showed an increase and decrease respectively in the long-term follow-up (Table 2). The comparisons between T0 and T1, T2, T3 and T4 for all variables analyzed in this study and the percentage of change, p-values, 95 % confidence intervals, and effect sizes are shown in the Supplementary Table S3.

### 3.3 Changes in biomarkers

BC survivors experienced significant reductions in most of the metabolic and hormonal biomarkers immediately after the 3 months of lifestyle intervention (i.e., glycemia, insulin, triglycerides, LDL, total cholesterol, and testosterone), significant decreases in glycemia and insulin were also observed up to 12-month follow-up time points (T1, T2, and T3), by contrast at 24-month (T4) values were only slightly reduced vs. T0. Another significant improvement is HOMA-IR index, which decreased in the short-term (T1) compared with baseline (T0), this improvement was also maintained after 6-month (T2) and up to 12-month (T3), but not in the long-term follow-up after 24-month (T4). In detail, the lifestyle intervention improves glycemic control: −8.51 % comparing T1 vs. T0 (p < 0.001), −10.02 % comparing T2 vs. T0 (p < 0.001), −5.08 % comparing T3 vs. T0 (p = 0.001) and −2.82 % comparing T4 vs. T0 respectively (no significant difference was found; p = 0.072). A similar effect was observed for insulin: −13.95 % T1 vs. T0 (p = 0.045), −21.29 % T2 vs. T0 (p = 0.002), −15.84 % T3 vs. T0 (p = 0.039), and −12.59 % T4 vs. T0 respectively (no significant difference was found; p = 0.082). About HOMA-IR index: −21.88 % T1 vs. T0 (p = 0.009), −29.60 % T2 vs. T0 (p = 0.001), −21.20 % T3 vs. T0 (p = 0.018), −17.26 % T4 vs. T0 (P = 0.062). Triglyceride levels decreased slightly at follow-up point T1 by −8.66 % (T1 vs. T0; p = 0.107), this decrease persists with significance at follow-up points T2 and T3 by −12.90 % (T2 vs. T0, p = 0.036) and −15.91 % (T3 vs. T0, p = 0.018) respectively, but did not continue up to 24-month of follow-up. Circulating LDL concentrations decreased significantly at T1 by −8.04 % (T1 vs T0, p = 0.001), but this significance did not persist at T2 and T3 follow-up. At T4, however, the decrease again and becomes significant by −6.06 % (T4 vs. T0, p = 0.049). It is notable a similar trend in total cholesterol (−4.19 % T1 vs. T0, p = 0.038; −3.71 % T2 vs. T0; −4.56 % T3 vs. T0, p = 0.043; −0.75 % T4 vs. T0). About testosterone: −35.39 % (T1 vs. T0, p = 0.001) and −23.55 % (T2 vs. T0, p = 0.007), but no significant changes in T3 and T4. The other parameters did not change during follow-up. The present study showed improvements in metabolic markers like glycemia, insulin, and HOMA-IR index. These trends were maintained up to the one-year follow-up. However, for the other hormonal and metabolic markers, only short term (e.g., testosterone) or unstable (e.g., LDL and total cholesterol) changes were measured over time.

The detailed comparison between T0 and other times for all biomarkers is shown in Table 3. The Supplementary Table 3S shows the comparisons between T0 and T1, T2, T3 and T4 for all variables analyzed in this study and the percentage of change, *p*-values, 95 % confidence intervals, and effect sizes.

**Table 3:** Comparison of prognostic biomarkers between T0 vs. T1, T2, T3 and T4 and percentage of change (% change) between T1, T2, T3 and T4 *vs* T0.

	Mean ± SD (T0)	Mean ± SD (T1)	Mean ± SD (T2)	Mean ± SD (T3)	Mean ± SD (T4)	% of change T1 vs T0	% of change T2 vs T0	% of change T3 vs T0	% of change T4 vs T0
**Glycemia (mg/dL)**	100.52 ± 11.93	91.97 ± 11.19***	90.45 ± 10.88***	95.41 ± 11.25**	97.6 ± 12.16	**−8.51 %**	**−10.02 %**	**−5.08 %**	−2.82 %
**Insulin (microU/mL)**	7.83 ± 4.80	6.74 ± 4.40*	6.16 ± 3.64**	6.59 ± 3.60*	6.84 ± 3.75	**−13.95 %**	**−21.29 %**	**−15.84 %**	−12.59 %
HOMA-IR index	2.04 ± 1.58	1.60 ± 1.26**	1.44 ± 1.47**	1.61 ± 1.08*	1.69 ± 1.06	**−21.88 %**	**−29.60 %**	**−21.20 %**	−17.26 %
**Triglycerides (mg/dL)**	103.17 ± 44.37	94.24 ± 41.35	89.86 ± 36.21*	86.76 ± 42.69*	94.83 ± 41.96	−8.66 %	**−12.90 %**	**−15.91 %**	−8.08 %
**HDL (mg/dL)**	62.62 ± 16.09	61.00 ± 13.71	60.62 ± 13.56	65.41 ± 12.82	64.38 ± 15.59	−2.59 %	−3.19 %	4.46 %	2.81 %
**LDL (mg/dL)**	136.00 ± 28.93	125.07 ± 28.22**	129.14 ± 27.33	129.55 ± 24.20	127.76 ± 23.65*	**−8.04 %**	−5.04 %	−4.74 %	**−6.06 %**
**Total cholesterol (mg/dL)**	216.45 ± 39.28	207.38 ± 37.43*	208.41 ± 37.20	206.59 ± 32.01*	214.83 ± 33.52	**−4.19 %**	−3.71 %	**−4.56 %**	−0.75 %
**Progesterone (ng/mL)**	0.49 ± 0.40	0.45 ± 0.19	1.16 ± 3.53	1.01 ± 3.31	0.71 ± 1.36	−6.12 %	138.78 %	106.12 %	44.90 %
**Testosterone (ng/mL)**	0.31 ± 0.26	0.20 ± 0.17**	0.24 ± 0.16**	0.27 ± 0.17	0.27 ± 0.17	**−35.39 %**	**−23.55 %**	−12.72 %	−14.77 %
**hs-Troponin (ng/L)**	2.97 ± 1.21	2.86 ± 2.80	2.93 ± 1.16	3.14 ± 1.95	2.66 ± 1.51	−3.70 %	−1.35 %	5.72 %	−10.44 %

Abbreviations: HDL, high-density lipoprotein; LDL, low-density lipoprotein; hs, high sensitive; T0, baseline; after 3 (T1); 6 (T2); 12 (T3), and 24 (T4) months after the lifestyle intervention; bold values denote statistical significance with a p < 0.05 (*); p < 0.01 (**); or p < 0.001 (***).

## 4 Discussion

This study continues the analysis of the impact of a 3-month home-based lifestyle intervention that focused on nutrition and exercise along with the pandemic emergency of COVID-19 in Italy, which enrolled non-physically active BC survivors with a high risk of recurrence [28]. In this previous study, beneficial effects of the LI were observed on Mediterranean diet adherence, and cardiometabolic parameters (pre vs post) [30]. Furthermore, the gut microbiota analysis showed a robust reduction of Proteobacteria after lifestyle intervention, which is able to reshape the gut microbiota by modulating microorganisms capable of decreasing inflammation and others involved in improving the lipid and glycemic assets of the host [45]. Specifically, in this study we followed the same cohort over the two-year pandemic period (from January 2020 to February 2022) and evaluated the effects of the intervention on lifestyle (modified compared to the original protocol) in the long term (2-year follow-up). In our study, we examined the changes related to anthropometrics, Mediterranean diet adherence, physical activity levels, cardiorespiratory fitness, and hormonal and metabolic biomarkers in the long term. The main finding of this study is that lifestyle intervention led to a cardiometabolic improvement not only in the first year of follow-up but also the long term (2-year follow-up). A singular result of the study is that the IA and CA showed similar changes over time in most of the analyzed parameters. This phenomenon could be explained by the changes to the original protocol imposed by the pandemic period. Indeed, it is likely that unlike similar populations who, in this period, have reduced levels of physical activity and worsened nutrition [46,47], the MoviS cohort has put the exercise and nutrition guidelines into practice, and at the same time, benefit from further feedback from experts, regardless of the original group to which they belonged. Particularly, the similar salutary effects in both arms were found not only immediately after the lifestyle intervention, as described by Natalucci et al. [28], but also after a short- (6-month) and long-term (12- and 24-month) follow-up. Indeed, the change in $\dot{V}$ O_2max_ show how cardiorespiratory fitness increases were maintained over time with a significant improvement for $\dot{V}$ O_2max_ values (increase of 11.21 % T1 vs. T0, 10.08 % T2 vs. T0, 7.19 % T3 vs. T0, and 7.04 % T4 vs. T0). The changes in Mediterranean diet adherence were also significant and persisted through follow-up points (+17.95 % T1 vs. T0, +22.62 % T2 vs. T0, +23.69 % T3 vs. T0 and +18.62 % T4 vs. T0). These effects agree with previous investigations for lifestyle modification strategies in BC survivors [20,[48], [49], [50], [51], [52], [53], [54], [55], [56]]. With the increasing trend of adopting remotely on-line technologies during this period of pandemic, the need for assessing a home-based lifestyle intervention, with a specific counselling on exercise recommendations and remotely supervised aerobic exercise, has become crucial and it turned out to be an effective intervention to improve the prognosis of the BC survivors [21,57]. A recent study also supports the hypothesis of the effectiveness of home-based exercise protocols [58]. The authors found no major differences in Metabolic equivalent of task (METs) or peak $\dot{V}$ O_2_ values recorded between pre- and post-pandemic in BC survivors who, due to the COVID-19 pandemic, were forced to continue the HEALTh exercise program (30 min of aerobic exercise at moderate intensity 3 d/week for 4 week) in March 2020. This suggests that the online modality may have served to keep the participants, who were otherwise likely to drop out of the HEALTh protocol, active. Virtual models such as this one just described could therefore help to preserve the necessary dose of physical activity to be able to prevent BC recurrence or assist its treatment [58]. Similar benefits in improving cardiorespiratory fitness in BC survivors have also been achieved using a high intensity interval training online program [59]. Some difficulties of online training (lack of adequate equipment and mobility restrictions) or in general lack of energy, fatigue, pain, worsening of the disease, lack of motivation and lack of support can be overcome with supervision. In fact, a supervisor, who is able to give specific guidance on exercise with specificity in terms of FITT-VP principle could be excellent strategies to encourage achievement of exercise guidelines [14,60]. The degree of involvement could also be increased with a cohesive group that, through technological means such as social chat, email, or video calls, could be supportive in understanding the discomforts associated with BC diagnosis or treatment [60]. Indeed, it is likely that the support received by our cohort of women resulted in the high level of adherence to exercise and nutrition guidelines even under extreme conditions. This adherence to the lifestyle change that the MoviS cohort of women has shown over time has also led to improvements in metabolic parameters such as glycemia (the decrease was −8.51 % T1 *vs.* T0, −10.02 % T2 *vs.* T0, −5.08 % T3 *vs.* T0 and −2.82 % T4 *vs.* T0), insulin (−13.95 % T1 *vs.* T0, −21.29 % T2 *vs.* T0, −15.84 % T3 *vs.* T0 and −12.59 % T4 *vs.* T0) and HOMA-IR index (−21.88 % T1 *vs* T0, −29.60 % T2 *vs* T0, −21.20 % T3 *vs* T0, −17.26 % T4 *vs* T0). In this line, it is also known that BC survivors’ risk of recurrence is associated with chronic inflammation, metabolic syndrome and physical inactivity [61,62]. Consequently, a low HOMA-IR index could be a good ally in preventing recurrence [63,64]. Moreover, the recent evidence associating insulin to BC recurrence is emerging and exercise and nutrition actions in the last decade have been recommended to modulate insulin levels [53,65]. Our findings are consistent with the epidemiological study Diet and Androgen-5 study (DIANA-5) [[63], [64], [65]], which found that BC survivors, who participated in a structured 3-month aerobic exercise program coupled with a Mediterranean diet, experienced improvements in their insulin levels, HOMA-IR index, and body composition parameters [66]. These results agree with other investigations which focused on health benefits of physical activity, healthy eating, and weight management for BC survivors [67]. Likewise, it is well known that the adherence to the Mediterranean diet is associated with a reduced BC risk and recurrence [16,68]. The nutritional choices inspired by the principles of the Mediterranean diet made by the BC survivors involved in this study go towards an improvement in eating habits (i.e., more adherent to the Mediterranean Diet). In this group of study, it is notable an increase in the consumption of dried fruit and cereal-based meals; a reduction of red meat, desserts and wine *per* week (data not shown). The improvement of MeDiet score obtained from the lifestyle intervention and maintained along the follow-up points (Table 2) is particularly interesting for the purposes of glycemic control, as highlighted by the amelioration of metabolic prognostic factors. Overweight, metabolic syndrome accompanied by sedentary behaviors have been established as notable risks for BC recurrence [69,70]. We also noted after lifestyle intervention a significant decrease of metabolic circulating factors such as triglycerides (−12.90 % T2 vs T0 and −15.91 % T3 *vs* T0), LDL (−8.04 % T1 *vs.* T0 and −6.06 % T4 *vs* T0), total cholesterol (−4.19 % T1 *vs.* T0 and −4.56 % T3 *vs* T0) and testosterone (−35.39 % T1 *vs.* T0 and −23.55 % T2 *vs* T0), whereas progesterone, hs-Troponin, and HDL did not show significant changes along 2-year follow-up.

The results obtained at the 2-year follow-up suggest that lifestyle intervention based on the motivational aspects and practical suggestions for exercise and nutrition habits significantly help to keep an adequate lifestyle in BC survivors. However, if adherence to the Mediterranean diet seems to be maintained after 2-year, the level of PA tends to decrease. This suggests that while nutritional support via the DianaWeb portal enabled women to continue to receive monthly feedback over the two years, supervision (on-site or remotely) with an exercise specialist limited to 3-month does not appear to be sufficient to support women in the long term.

### 4.1 Strengths and limitations

The main strengths of this work were the importance of timeliness with assessments, the long-term follow-up of 2-year, and the comprehensiveness of clinical and functional outcomes. Indeed, one important finding is the duration of the effects obtained in terms of improvement of lifestyle cardiometabolic related risk factors and consequent prognostic advantage. Furthermore, the long-term follow-up data will provide important information to optimize a personalized educational intervention.

However, our study has some limitations. The first is the small sample size, it would be interesting to evaluate the same results in larger and prospective trials with patients enrolled from multiple center. The second is the absence of a control group and the third is linked to the pandemic period and unpredictable variables such as mental health within the home-confinement due to the 2-year of the COVID-19 emergency.

## 5 Conclusions

Despite the COVID-19 pandemic, our results show how a 3-month lifestyle intervention – with a mixed approach (on-site and remotely) – focused on Mediterranean diet and aerobic training, significantly improved cardiometabolic outcomes in the long term (2-year follow-up) in BC survivors.

In light of these preliminary results, the challenge for a further therapeutic care guideline for BC survivors is to provide complementary support to the therapies with the aim of reducing the risk of relapse and healthcare burder. This modeling approach with the prescription of a healthy lifestyle confirms the efficacy of a multidisciplinary team (oncologists, nutritionists, exercise specialists, and biochemists/molecular biologists) in reducing the barriers present in this vulnerable population and in promoting the awareness of the benefits induced by exercise and nutritional interventions.

The take-home message of this work in the scientific community is: ‘Lifestyle medicine for BC survivors is a coadjutant to cancer treatment, improves the cardiometabolic conditions, powerful for prognostic advantage. Nowadays, it is necessary to spread this model on health and wellness beyond care, providing synergistic involvement among sectors and actors that have capacity to perform on socioeconomic determinants and allow the prescription of healthy lifestyles in clinical practices’.

## Funding

This research was supported in part by a grant from the Athenaeum of Urbino entitled: Promozione della salute e della sicurezza alimentare (DR. n. 226/2021 19/05/2021). The funders had no role in study design, data collection and analysis, decision to publish, or preparation of the manuscript.

## Institutional review board statement

The study followed the international ethical recommendations contained in the Declaration of Helsinki and received ethical approval from the local Ethics Committee (permission number: 21/19 10. July 2019. This trial is registered on ClinicalTrials gov: NCT04818359).

## Informed consent statement

Informed consent was obtained from all subjects who participated in the study.

## Data availability statement

Data will be made available on request.

## CRediT authorship contribution statement

**Valentina Natalucci:** Supervision, Methodology, Investigation, Data curation. **Carlo Ferri Marini:** Writing – review & editing, Supervision, Formal analysis, Data curation. **Francesco Lucertini:** Methodology, Investigation, Formal analysis, Conceptualization. **Giosuè Annibalini:** Writing – review & editing, Methodology, Conceptualization. **Davide Sisti:** Software, Methodology, Data curation. **Luciana Vallorani:** Formal analysis, Data curation. **Roberta Saltarelli:** Formal analysis, Data curation. **Andrea Rocco Panico:** Data curation. **Marta Imperio:** Writing – review & editing, Formal analysis, Data curation. **Marco Flori:** Methodology, Formal analysis. **Paolo Busacca:** Formal analysis. **Anna Villarini:** Supervision, Conceptualization. **Sabrina Donati Zeppa:** Writing – review & editing, Methodology. **Deborah Agostini:** Writing – review & editing, Formal analysis. **Silvia Monaldi:** Data curation. **Simone Barocci:** Software, Resources, Methodology, Investigation, Data curation, Conceptualization. **Vincenzo Catalano:** Resources, Methodology. **Marco Bruno Luigi Rocchi:** Supervision. **Piero Benelli:** Resources, Formal analysis. **Vilberto Stocchi:** Visualization, Supervision. **Elena Barbieri:** Writing – review & editing, Writing – original draft, Supervision, Project administration, Funding acquisition, Conceptualization. **Rita Emili:** Writing – review & editing, Writing – original draft, Resources, Project administration, Investigation, Funding acquisition, Formal analysis, Conceptualization.

## Declaration of competing interest

The authors declare that they have no known competing financial interests or personal relationships that could have appeared to influence the work reported in this paper.
