# Supplementary material for: Effect of a lifestyle intervention program's on breast cancer survivors' cardiometabolic health: Two-year follow-up
Source: Heliyon. 2023 Oct 29;9(11):e21761. doi: 10.1016/j.heliyon.2023.e21761 (PMC10651516; doi:10.1016/j.heliyon.2023.e21761)
Supplement: Multimedia component 1 [file mmc1.docx]

**Supplementary Table S1.** Comparison of anthropometric and body composition parameters, cardiorespiratory fitness, physical activity level, and dietary habits after a 12-week intervention (T1) between a Control Arm and an Intervention Arm.

|  | **Control arm**  **Mean ± SD (T1)** | **Intervention arm**  **Mean ± SD (T1)** |  | **P(t)** |  |  |
| --- | --- | --- | --- | --- | --- | --- |
| **Body weight (kg)** | 63.79±8.91 | 68.89±12.33 |  | 0.139 |  |  |
| **BMI (kg/m^2^)** | 24.56±3.93 | 26.47±5.23 |  | 0.231 |  |  |
| **Waist Circumference (cm)** | 81.83±10.02 | 87.39±11.01 |  | 0.140 |  |  |
| **Fat mass (%)** | 30.20±5.45 | 31.25±6.47 |  | 0.642 |  |  |
| ***V̇O_2max_* (mL·min^−1^·kg^−1^)** | 34.01±6.3 | 33.61±7.0 |  | 0.915 |  |  |
| **PAL (MET-min/week)** | 394.6±385.53 | 291.4±214.54 |  | 0.417 |  |  |
| **Adherence to Mediterranean diet**  **(MeDiet Score DianaWeB)** | 8.47±2.29 | 7.4±1.5 |  | 0.142 |  |  |
| **Glycemia (mg/dL)** | 89.93±9.12 | 93.33±12.97 |  | 0.532 |  |  |
| **Insulin (microU/mL)** | 5.94±3.74 | 6.55±3.86 |  | 0.660 |  |  |
| **HOMA-IR index (mg/dl)** | 1.41±1.08 | 1.76±1.31 |  | 0.440 |  |  |
| **Triglycerides (mg/dL)** | 86.29±50.84 | 100.20±35.59 |  | 0.431 |  |  |
| **HDL (mg/dL)** | 61.33±11.70 | 60.20±15.56 |  | 0.895 |  |  |
| **LDL (mg/dL)** | 123.33±33.72 | 128.87±22.49 |  | 0.660 |  |  |
| **Total cholesterol (mg/dL)** | 205.93±41.53 | 211.00±33.74 |  | 0.734 |  |  |
| **Progesterone (ng/mL)** | 0.45±0.23 | 0.52±0.19 |  | 0.448 |  |  |
| **Testosterone (ng/mL)** | 0.16±0.16 | 0.28±0.21 |  | 0.097 |  |  |
| **hs-Troponin (ng/L)** | 2.14±1.10 | 3.36±3.79 |  | 0.298 |  |  |

Abbreviations: BMI, body mass index; *V̇*O_2max_, maximal oxygen uptake; PAL, physical activity level; HOMA-IR, homeostasis model assessment-insulin resistance; HDL, high-density lipoprotein; LDL, low-density lipoprotein; hs, high sensitive.
