# Supplementary material for: Effect of a lifestyle intervention program's on breast cancer survivors' cardiometabolic health: Two-year follow-up
Source: Heliyon. 2023 Oct 29;9(11):e21761. doi: 10.1016/j.heliyon.2023.e21761 (PMC10651516; doi:10.1016/j.heliyon.2023.e21761)
Supplement: Multimedia component 2 [file mmc2.docx]

| **MEDIET modified questionnaire** |
| --- |
| Code pt. (WEB CODE) |
| Time frame (T0, T1, T2, T3, T4) |
| Time |
| Date |
| Questions related to daily consumption |
| Use of olive oil as the main culinary fat |
| Consumption of ≥ 4 tablespoons/d of olive oil (including oil used for frying, salads, out-of-house meals, etc.) |
| Consumption of ≥ 3 servings/d of fruits (1 serving=100 - 150 g) |
| Consumption of ≥ 3 servings/week of dried fruits (including nuts, almonds, peanuts etc.) (1 serving=30 g which correspond to 7 unshelled walnuts or 10 almonds) |
| Consumption of ≥ 2 servings/d of vegetables (carrots, zucchini, tomatoes, etc. excluding potatoes and legumes) (1 serving=200 g) |
| Consumption of potatoes per week (1 serving=150 g circa (weight before cooking) |
| Consumption of white bread per day (1 serving=70 g) |
| Consumption of whole wheat bread per day (1 serving=70 g) |
| Consumption of white pasta per week (1 serving=70 g) |
| Consumption of whole wheat pasta per week (1 serving=70 g) |
| Consumption of refined (white) rice per week (1 serving=60 g) |
| Consumption of brown or basmati rice per week (1 serving=70 g) |
| Consumption of other grains (spelt, kamut, barley...) per week (1 serving=70 g) |
| Consumption < 1 serving/d of sweetened and/or carbonated beverages (1 serving= |
| 330 ml) |
| Consumption of < 1 serving/d of butter, margarine, or cream (1 serving=12 g) |
| Consumption of cheese per week (1 serving=70 g) |
| Consumption of grana cheese as a condiment per week, e.g. to season pasta (1 serving=10 g) |
| Consumption of cow's or goat's milk per week. Is the milk consumed whole, part-skim or skim (1 serving=150 ml) |
| Consumption of yogurt per week (1 serving=125 ml) |
| Consumption of < 1 serving/d of red meat, hamburger or meat products (ham, sausage, etc.) (1 serving=150 g) |
| Consumption of preserved meat (various cold cuts or sausages) per week (1 serving=150 g) |
| Preferential consumption of chicken, turkey or rabbit meat instead of veal, pork, hamburger or sausage (1 serving=150 g) |
| Consumption of eggs (including as ingredients) per week (1 serving=50 g) |
| Consumption of ≥ 3 servings/week of legumes (1 serving=40 g (dry weight), 120 g, weight after cooking) |
| Consumption of ≥ 3 servings/week of fish or shellfish (1 serving=150 g of fish or 150 g of seafood or shellfish, weight before cooking) |
| Consumption of < 3 servings/week of commercial sweets or pastries (not homemade), such as cakes, cookies, biscuits or custard 1 serving=3 cookies or a small slice of cake or a small ice cream |
| Consumption of ≥ 1 serving/d of wine (1 serving=125 ml) |
| Consumption of super alcohol per week (1 serving=45 ml) |
