# Supplementary material for: Effect of a lifestyle intervention program's on breast cancer survivors' cardiometabolic health: Two-year follow-up
Source: Heliyon. 2023 Oct 29;9(11):e21761. doi: 10.1016/j.heliyon.2023.e21761 (PMC10651516; doi:10.1016/j.heliyon.2023.e21761)
Supplement: Multimedia component 3 [file mmc3.docx]

**Supplementary Table S3.** Comparison between T0 vs. T1, T2, T3 and T4 for all variables analysed in this study and percentage of change, *p*-values, 95% confidence intervals, and effect sizes.

|  |  | Mean difference | 95% CI_inf_ | 95% CI_sup_ | % of change | *p*-values | Effect size (partial h^2^) |
| --- | --- | --- | --- | --- | --- | --- | --- |
| **Body weight (kg)** | T1 vs T0 | 0.762 | −0.182 | 1.706 | −1.13% | 0.109 | 0.089 |
|  | T2 vs T0 | 0.871 | −0.231 | 1.972 | −0.70% | 0.117 | 0.086 |
|  | T3 vs T0 | 0.145 | −1.155 | 1.445 | −0.21% | 0.821 | 0.002 |
|  | T4 vs T0 | −0.576 | −2.042 | 0.891 | 0.85% | 0.428 | 0.023 |
| **BMI (kg/m^2^)** | T1 vs T0 | **0.479** | **0.044** | **0.915** | **−1.83%** | **0.032** | **0.154** |
|  | T2 vs T0 | 0.097 | −0.381 | 0.574 | −0.37% | 0.682 | 0.006 |
|  | T3 vs T0 | 0.169 | −0.386 | 0.724 | −0.65% | 0.538 | 0.014 |
|  | T4 vs T0 | −0.072 | −0.68 | 0.535 | 0.28% | 0.809 | 0.002 |
| **Waist Circumference (cm)** | T1 vs T0 | 0.357 | −1.606 | 2.320 | 0.30% | 0.712 | 0.005 |
|  | T2 vs T0 | **2.955*** | **1.229** | **4.681** | **−3.46%** | **0.002** | **0.305** |
|  | T3 vs T0 | −0.638 | −3.054 | 1.778 | 0.75% | 0.593 | 0.010 |
|  | T4 vs T0 | −0.283 | −2.683 | 2.118 | 0.33% | 0.811 | 0.002 |
| **Fat mass (%)** | T1 vs T0 | 0.61 | −0.25 | 1.471 | −2.81% | 0.157 | 0.070 |
|  | T2 vs T0 | −0.038 | −0.937 | 0.861 | 0.17% | 0.932 | 0.000 |
|  | T3 vs T0 | −0.245 | −1.300 | 0.811 | 1.13% | 0.638 | 0.008 |
|  | T4 vs T0 | **−1.324*** | **−2.555** | **−0.094** | **6.10%** | **0.036** | **0.148** |
| ***V̇ O_2max_* (mL·min**^−1^**·kg**^−1^**)** | T1 vs T0 | **−3.421*** | **−4.400** | **−2.441** | **11.21%** | **<0.001** | **0.646** |
|  | T2 vs T0 | **−3.379*** | **−4.787** | **−1.972** | **11.08%** | **<0.001** | **0.463** |
|  | T3 vs T0 | **−2.193*** | **−3.474** | **−0.912** | **7.19%** | **0.002** | **0.305** |
|  | T4 vs T0 | **−2.148*** | **−3.918** | **−0.378** | **7.04%** | **0.019** | **0.181** |
| **PAL (MET-min/week)** | T1 vs T0 | −0.028 | −0.106 | 0.05 | 1.93% | 0.475 | 0.018 |
|  | T2 vs T0 | 0.052 | −0.025 | 0.128 | −3.72% | 0.177 | 0.064 |
|  | T3 vs T0 | −0.017 | −0.067 | 0.033 | 1.22% | 0.485 | 0.018 |
|  | T4 vs T0 | **.062*** | **0.004** | **0.12** | **−4.51%** | **0.036** | **0.147** |
| **Adherence to Mediterranean diet (MeDiet Score DianaWeB)** | T1 vs T0 | **−1.207** | **−1.794** | **−0.62** | **17.95%** | **<0.001** | **0.388** |
|  | T2 vs T0 | **−1.521** | **−2.237** | **−0.804** | **22.62%** | **<0.001** | **0.403** |
|  | T3 vs T0 | **−1.593** | **−2.225** | **−0.961** | **23.69%** | **<0.001** | **0.488** |
|  | T4 vs T0 | **−1.252** | **−1.989** | **−0.514** | **18.62%** | **0.002** | **0.301** |
| **Glycemia (mg/dL)** | T1 vs T0 | **8.552*** | **5.354** | **11.750** | **−8.51%** | **<0.001** | **0.517** |
|  | T2 vs T0 | **10.069*** | **7.243** | **12.895** | **−10.02%** | **<0.001** | **0.655** |
|  | T3 vs T0 | **5.103*** | **2.373** | **7.834** | **−5.08%** | **0.001** | **0.344** |
|  | T4 vs T0 | 2.828 | −0.268 | 5.923 | −2.82% | 0.072 | 0.111 |
| **Insulin (microU/mL)** | T1 vs T0 | **1.093*** | **0.028** | **2.159** | **−13.95%** | **0.045** | **0.136** |
|  | T2 vs T0 | **1.669*** | **0.67** | **2.668** | **−21.29%** | **0.002** | **0.295** |
|  | T3 vs T0 | **1.241*** | **0.068** | **2.415** | **−15.84%** | **0.039** | **0.144** |
|  | T4 vs T0 | 0.986 | −0.134 | 2.106 | −12.59% | 0.082 | 0.104 |
| **HOMA-IR index** | T1 vs T0 | **0.449** | **0.122** | **0.775** | **−21.88%** | **0.009** | **0.220** |
|  | T2 vs T0 | **0.607** | **0.289** | **0.924** | **−29.60%** | **0.001** | **0.354** |
|  | T3 vs T0 | **0.435** | **0.079** | **0.79** | **−21.20%** | **0.018** | **0.183** |
|  | T4 vs T0 | 0.354 | −0.019 | 0.727 | −17.26% | 0.062 | 0.119 |
| **Triglycerides (mg/dL)** | T1 vs T0 | 8.931 | −2.057 | 19.919 | −8.66% | 0.107 | 0.090 |
|  | T2 vs T0 | **13.310*** | **0.926** | **25.695** | **−12.90%** | **0.036** | **0.148** |
|  | T3 vs T0 | **16.414*** | **3.075** | **29.752** | **−15.91%** | **0.018** | **0.185** |
|  | T4 vs T0 | 8.345 | −5.758 | 22.448 | −8.08% | 0.236 | 0.050 |
| **HDL (mg/dL)** | T1 vs T0 | 1.621 | −1.159 | 4.400 | −2.59% | 0.242 | 0.048 |
|  | T2 vs T0 | 2.000 | −0.624 | 4.624 | −3.19% | 0.130 | 0.080 |
|  | T3 vs T0 | −2.793 | −5.784 | 0.197 | 4.46% | 0.066 | 0.116 |
|  | T4 vs T0 | −1.759 | −4.676 | 1.159 | 2.81% | 0.227 | 0.052 |
| **LDL (mg/dL)** | T1 vs T0 | **10.931*** | **5.179** | **16.683** | **−8.04%** | **0.001** | **0.351** |
|  | T2 vs T0 | 6.862 | −0.237 | 13.961 | −5.04% | 0.058 | 0.123 |
|  | T3 vs T0 | 6.448 | −0.274 | 13.171 | −4.74% | 0.059 | 0.121 |
|  | T4 vs T0 | **8.241*** | **−0.057** | **16.539** | **−6.06%** | **0.049** | **0.129** |
| **Total cholesterol (mg/dL)** | T1 vs T0 | **9.069*** | **0.533** | **17.605** | **−4.19%** | **0.038** | **0.145** |
|  | T2 vs T0 | 8.034 | −2.078 | 18.147 | −3.71% | 0.115 | 0.086 |
|  | T3 vs T0 | **9.862*** | **0.317** | **19.407** | **−4.56%** | **0.043** | **0.138** |
|  | T4 vs T0 | 1.621 | −10.261 | 13.503 | −0.75% | 0.782 | 0.003 |
| **Progesterone (ng/mL)** | T1 vs T0 | 0.032 | −0.121 | 0.186 | −6.12% | 0.669 | 0.007 |
|  | T2 vs T0 | −0.676 | −2.036 | 0.685 | 138.78% | 0.318 | 0.036 |
|  | T3 vs T0 | −0.52 | −1.661 | 0.621 | 106.12% | 0.359 | 0.030 |
|  | T4 vs T0 | −0.219 | −0.622 | 0.184 | 44.90% | 0.275 | 0.042 |
| **Testosterone (ng/mL)** | T1 vs T0 | **.112*** | **0.053** | **0.171** | **−35.39%** | **0.001** | **0.353** |
|  | T2 vs T0 | **.075*** | **0.022** | **0.128** | **−23.55%** | **0.007** | **0.229** |
|  | T3 vs T0 | −1.564 | −4.224 | 1.096 | −12.72% | 0.141 | 0.076 |
|  | T4 vs T0 | 0.047 | −0.008 | 0.102 | −14.77% | 0.091 | 0.099 |
| **hs-Troponin (ng/L)** | T1 vs T0 | 0.103 | −0.852 | 1.059 | −3.70% | 0.826 | 0.002 |
|  | T2 vs T0 | 0.034 | −0.403 | 0.472 | −1.35% | 0.873 | 0.001 |
|  | T3 vs T0 | −0.172 | −0.87 | 0.525 | 5.72% | 0.617 | 0.009 |
|  | T4 vs T0 | 0.31 | −0.168 | 0.788 | −10.44% | 0.194 | 0.059 |

Abbreviations: inferior (CI_inf_) and superior (CI_sup_) confidence interval; BMI, body max index; *V̇*O_2max_, maximal oxygen uptake; PAL, physical activity level; HOMA-IR, homeostasis model assessment-insulin resistance; HDL, high-density lipoprotein; LDL, low-density lipoprotein; hs, high sensitive T0, baseline; T1, after 3 months; T2, after 6 months; T3, after 12 months; T4, after 24 months.
